# Ague as a Prophylactic of Phthisis

**Published:** 1884-12

**Authors:** 


**Ague as a Prophylactic of Phthisis.**—The *London Medical Record* says: This action of ague is relative rather than positive; that is, phthisis occurs more frequently in regions where intermittents are not epidemic, and *vice versa*; we cannot say that, where intermittents are dominant, there phthisis is not met with—both diseases may occur simultaneously in the same person. Dr. Vieta (*El Genio Medico-Quirurgical*, January 15, 1883) describes his experience in Azagra, in the kingdom of Navarre. The situation of the town is damp and low; it is surrounded by the rivers Ebro and Ega, and formerly these constantly overflowed their banks, submerging half the town; the streets were unpaved, and full of holes, in which the water lodged. In the outskirts much hemp was cultivated, which, when cut, was macerated in pools, of which there were a very large number. Intermittent fever in its worst forms was very prevalent. Now the streets are paved, the rivers embanked so that they no longer overflow, and hemp is not so much grown, market-gardening being found more profitable. Intermittents are no longer endemic; a few simple cases occur, but the paludal cachexie is no more seen, and the cases yield readily to treatment. But with this diminution of intermittents there is a marked increase in the number of chronic affections of the chest, and especially of phthisis, which was formerly almost unknown. Dr. Vieta does not attempt to account for this antagonism; he attributes the phthisis to neglected bronchial catarrh, and not to hereditary influence or diathesis, which does not seem to exist. He therefore hopes that improved hygienic knowledge and practice, leading the people to be more careful as to exposure, etc., will diminish the number of cases of pulmonary disease.

**Urinary Test Papers.**—Messrs. Parke, Davis & Co., of Detroit, Michigan, have published a very excellent and valuable paper, in which is detailed, with great care and fidelity, the method for using the urinary test papers introduced to the profession by Dr. G. Oliver. Dr. Oliver's methods have been extensively adopted in Europe, and have given great satisfaction. The color scale used in connection with this method may be obtained by addressing Messrs. Parke, Davis & Co., who deserve the thanks of the physicians of this country for the trouble and expense incurred by them in connection with this interesting subject.

**The Therapeutic Properties of Buttermilk.**—Buttermilk, so generally regarded as a waste product, has latterly been coming somewhat into vogue not only as a nutrient, but as a therapeutic agent, and in an editorial article the *Canada Lancet*, some time ago, highly extolled its virtues. Buttermilk may be roughly described as milk which has lost most of its fat and a small percentage of its casein, and which has become sour by fermentation. Long experience has demonstrated it to be an agent of superior digestibility. It is, indeed, a true milk peptone, that is, milk already partially digested, the coagulation of coagulable portion being loose and flaky, and not of that firm indigestible nature which is the result of the action of the gastric juice upon sweet cow's milk. It resembles koumiss in its nature, and, with the exception of that article, it is the most grateful, refreshing and digestible of the products of milk. It is a decided laxative to the bowels, a fact which must be borne in mind in the treatment of typhoid fever, and which may be turned to advantage in the treatment of habitual constipation. It is a diuretic, and may be prescribed with advantage in some kidney troubles. Owing to its acidity, combined with its laxative properties, it is believed to exercise a general impression on the liver. It is well adapted to many cases where it is customary to recommend lime-water and milk. It is invaluable in the treatment of diabetes, either exclusively or alternating with skimmed milk. In some cases of gastric ulcer, and cancer of the stomach, it is the only food that can be retained.—*Medical Journal.*

**Menthol.**—This substance has recently been brought into notice as a remedy for headache and neuralgia. It is a white, semi-crystalline body, with a strong, burning odor of peppermint, and is usually made into small cones mounted on a woodan handle, and sold under the name of "Pain pencil."

If it is rubbed over the locality of a headache or other pain; a burning sensation is first felt, followed by a feeling of refreshing coolness, and temporary relief of the pain.

The liquid oil of Japanese peppermint has long heen used in Japan and China for this purpose; and travelers have frequently brought home small vials of this oil, inscribed with oriental heiroglyphics, and warranted to be a sure destroyer of pain.

Menthol is simply the solid constituent of this oil. Chemically considered, it is a camphor, and has the formula $C_{10}H_{20}O$, differing only from ordinary camphor ($C_{10}H_{16}O$) by the addition of four atoms of hydrogen. In the liquid oil of peppermint it exists in solution with a liquid hydrocarbon of the turpentine series $C_{10}H_{16}$, but can be readily crystalized out. Its medicinal effects are probably due to the counter-irritation it sets up; but the strong, agreeable odor of peppermint may also have some effect on the nerves. Although the relief it gives is generally only temporary, yet it is certainly harmless, and may prove a valuable addition to the list of popular remedies.

**Sneezing and Shivering.**—Nature's provisions against the consequences of a "chill," and the prevention of a "cold," are sneezing and shivering. A violent fit of sneezing often saves a chilled body the consequences of the nerve depression or "shock" to which it has been subjected, and this shock may in its first impression be very limited in its area ; for example, the small extent covered by a draught of cold air rushing through the crevice of a door or window. The nerve centers are roused from their "collapse" by the commotion or explosive influence of the sneeze. If sneezing fails, nature will try a shiver, which acts mechanically in this way. If this fails, the effects are likely to be very serious, and bad consequences may ensue. The cold is slight when sneezing suffices to recover the nervous system quickly from its depression, and grave when even strong shivering fails to do so.

There is poetry and there is beauty in real sympathy; but there is more—there is *action*. The noblest and most powerful form of sympathy is not merely the responsive tear, the echoed sigh, the answering look, it is the embodiment of the sentiment in *actual* help.

